# Taking a Community-Partnered Approach to Developing Culturally-Responsive Mental Health Screening Materials for African-Born Adults in the United States

**DOI:** 10.3390/bs16060993

**Published:** 2026-06-15

**Authors:** Anu Asnaani, Tatiana Leroy, Valentine Mukundente, Jackson Webb Hunter, Jacqueline Kent-Marvick, Sara E. Simonsen

**Affiliations:** 1 Department of Psychology, University of Utah, Salt Lake City, UT 84112, USA; 2 Best of Africa Organization, Salt Lake City, UT 84128, USA; 3 College of Nursing, University of Utah, Salt Lake City, UT 84112, USA

**Keywords:** African, anxiety, culturally-responsive, immigrant, mental health screening, refugee, stigma

## Abstract

Despite a large number of African-born individuals residing in the United States, there is a significant disparity in how this community accesses and utilizes mental health treatment. Low screening rates for common mental health concerns is one crucial part of ongoing inequities in mental healthcare access. Willingness to engage in screening is negatively impacted by a lack of culturally responsive ways to make screening more acceptable and stigma with mental health. This study therefore aimed to examine the perceived acceptability and utility of community-developed patient vignettes created to increase willingness to be screened for common mental health concerns. Employing a qualitative approach, a community advisory board (CAB) (*n* = 5) was enlisted to co-develop vignettes outlining an African community member’s symptoms of anxiety and subsequent help-seeking behavior. Two focus groups of community members (*n* = 18) provided qualitative feedback on the vignettes and shared their general attitudes towards mental health and recommendations for mental health screening and treatment in the African community. Using a hybrid inductive and deductive qualitative descriptive approach and classifying responses based on the socioecological model, four major themes emerged from the data: (1) between support and strain: the role of family; (2) reducing stigma: community voices as education; (3) culture as a barrier and a bridge; and (4) the importance of stories that reflect lived experience. Overall, participants were receptive to the culturally-responsive mental health vignettes and provided fruitful suggestions for how these stories can be used to reduce stigma and increase willingness to seek screening and treatment in African-born residents of the United States.

## 1. Introduction

As of 2019, there were more than 2 million sub-Saharan African-born individuals residing in the United States (U.S; Lorenzi & Batalova, 2022). Mental health burden is a significant concern among these sub-Saharan African residents of the U.S. (Venters et al., 2011). Prevalence data is limited for this community, but mental health problems are disproportionately experienced by African immigrants when compared to the general U.S. adult population, with nationally sampled epidemiological datasets finding 34.6% of those identifying as African migrants experiencing anxiety versus 19.1% of U.S. adults (James et al., 2022), and 37.9% experiencing post-traumatic stress disorder ([PTSD] versus 8 to 15% of the general population; Kessler et al., 2005). In addition, a systematic review with an accompanying meta-analysis has found that 33.2% of African migrants surveyed report experiencing depression versus 8.1% of the general population in the U.S. (Brody et al., 2018). These high levels of mental health burden are likely due in part to traumatic experiences prior to forced migration (e.g., political and economic instability) and resettlement-related stress (Boise et al., 2013; Sellers et al., 2006).

Given this documented higher mental health burden for this subgroup of immigrants in Western contexts, addressing barriers to mental health screening and care utilization is relevant to promoting mental health equity (i.e., ensuring all individuals have the ability to achieve maximal levels of mental wellness) in this largely forcibly displaced population, given the documented deleterious impact of deficient screening rates on mental healthcare utilization and overall mental healthcare burden for other refugee populations (e.g., Magwood et al., 2022; Polcher & Calloway, 2016). While limited research has been conducted on strategies to promote mental health screening and healthcare utilization in African immigrants specifically, it is conceivable that a lack of mental health screening contributes to mental health inequity (i.e., systematic and pervasive differences in mental health disorder prevalence and treatment utilization, as discussed previously) in African-born immigrants who are currently residents in the United States. Indeed, we know that despite the recommendation for routine screening and the known greater burden of mental health problems, African immigrants utilize these services at lower rates than their non-immigrant counterparts (Centers for Disease Control and Prevention, 2022; Derr, 2016).

Thus, mental health screening remains a priority in order to address the needs of African immigrants or refugees residing in the U.S., but it is not happening enough in healthcare settings. Healthy People 2030 has a goal that 13.5% of primary care visits with adolescents and adults include screening for depression, as data indicate that only 9.1% of primary care visits include screening for depression (Office of Disease Prevention and Health Promotion, n.d.). Further, the United Nations has outlined 17 Sustainable Development Goals (UN General Assembly, 2015) to ensure prosperity for communities and countries across the globe, with the goals of “good health and wellbeing”, “reduced inequalities” and “partnerships for the goals” (in this case community partnerships) being particularly relevant to the current study’s focus of improving screening rates for mental health disorders for African-born adults. In contrast to both these sets of guidelines/goals, we have found in our own research that African immigrants were 73% less likely to have a regular healthcare provider than were otherwise similar African American women (Ahad et al., 2019), impacting their ability to receive mental health screening in primary care settings. In addition, other previous work with this community has found that African-born residents of the U.S. are much more reticent to seek mental health treatment even in comparison to other immigrant or racially diverse groups (e.g., Latinos, Pacific Islanders, or African Americans; Gutierrez Chavez et al., 2022), and there is a strong preference conveyed by African community members for mental health providers who share similar cultural backgrounds or are already trusted stewards within the community (Kaur et al., 2022). Taken together, promoting and implementing mental health screening in community settings with community health workers (CHWs) who are already members of the community is an ideal way to increase screening rates and mental healthcare utilization, and to appreciably address ongoing mental health disparities for this community.

As they adjust to life in the U.S., African-born adults also face multiple unique challenges compared to non-immigrant subgroups, including language barriers (e.g., Adekeye et al., 2014; Omenka et al., 2020), unfamiliarity with available social and medical services (e.g., Boise et al., 2013), social isolation and loneliness (e.g., Escamilla & Saasa, 2020; Saasa & Miller, 2021), discrimination and racism (e.g., Adekeye et al., 2014; Saechao et al., 2012), and a lack of culturally competent service providers (e.g., Adekeye et al., 2014; Boise et al., 2013). As noted by Boise et al. (2013), African immigrants often perceive the U.S. healthcare system as confusing and services as difficult to access, resulting in the overuse of emergency care. Additionally, because U.S. healthcare providers often lack understanding of African perspectives on health and illness, opportunities are missed to provide patient-centered care and to address root causes of mistrust in the healthcare setting. Compounding the issue of mental health for African immigrants are poor accessibility and underutilization of mental health services (e.g., Ahad et al., 2019; Ayele et al., 2020; Derr, 2016; Venters et al., 2011). Service-use data are extremely limited. The sparse available evidence indicates that relatively few African immigrants (approximately 5–13%) access formal mental healthcare services (e.g., Ayele et al., 2020; Fenta et al., 2006). By contrast, a larger proportion (approximately 8–17%) seek support from non-healthcare sources such as religious leaders, family, and traditional healers (Ayele et al., 2020; Fenta et al., 2006). These inequities are further exacerbated by the current sociopolitical context in which refugee programs have been discontinued in the United States, with a number of refugee-related health services (including those for African-born refugees) no longer receiving funding for resettlement services (Rfat et al., 2025).

Despite these significant, multifactorial structural and contemporary barriers to screening and mental healthcare utilization in African immigrants, it is important to recognize that some factors are modifiable. For example, stigma related to mental health experienced among this group is a leading barrier to the use of services, as evident from multiple large-scale and local mixed methods studies (e.g., Derr, 2016; Gutierrez Chavez et al., 2022); however, as compared to structural barriers, this is a modifiable factor. Mental health knowledge, which has also been consistently raised by this community as a barrier to seeking mental health services (Kaur et al., 2022; Gutierrez Chavez et al., 2022), is another factor that can be addressed through psychoeducation (Pederson et al., 2022, 2023). Remaining factors such as language barriers, mistrust of the healthcare system, concerns about discrimination and racism, and a lack of culturally competent healthcare providers can potentially be ameliorated with the use of CHWs, thereby providing a multi-faceted approach to addressing key barriers to treatment utilization for those community members that are in need of such health-promoting services. These points illustrate that addressing the unique cultural beliefs, values, and other social determinants of health (SDOH) that serve as barriers to mental health screening and care utilization among African immigrants is essential for increasing mental healthcare uptake in this population (e.g., Derr, 2016; Omenka et al., 2020).

The foundation for this study is based on a community-identified need to promote mental health screening and referral in African-born community members, based on studies within and with these communities by our authorship team. Our work is rooted in our long-standing partnerships with Best of Africa (e.g., Ahad et al., 2019; Asnaani et al., 2022; Simonsen et al., 2015), a community organization based in Utah that is founded by Sub-Saharan African immigrants to address health disparities facing this population. In a previous community-engaged randomized trial of a wellness coaching intervention delivered by CHWs which included 84 African immigrants (e.g., Buder et al., 2018; Simonsen et al., 2015), we identified an unmet need for mental healthcare; 34% of African immigrant women screened positive for depression but most declined follow-up care. CHWs posited that mental healthcare was not prioritized due to stigma and other systemic and community-level barriers to care-seeking. This was further confirmed by our subsequent mixed-methods study with Best of Africa, revealing that screenings for depression, anxiety, and PTSD were particularly high priorities of unmet need, and that barriers including stigma, lack of culturally competent providers, cost, and lack of awareness about mental health perpetuated this disparity for this community (e.g., Asnaani et al., 2022; Kaur et al., 2022). Moreover, community members from Best of Africa communicated a need to address multiple barriers to mental health services, further underscoring the need for multilevel interventions that address the unique barriers in this community to improve access and utilization of mental health services.

Indeed, this expressed need by our local African partners reflects a wider finding in the literature that stigma presents a particularly problematic barrier to seeking or obtaining mental healthcare, which is an issue for refugees and immigrants more broadly in Western contexts (Yılmaz et al., 2025; Ambrose et al., 2025; Nickerson et al., 2020) and which deserves particular attention to be addressed adequately. While work to address these barriers has been sparse, some scholars have approached it through exploration of more comprehensive mental health measure development (e.g., Kira et al., 2012), and use of stories and narratives (i.e., mental health vignettes) from real or fictional community members to reduce stigma in immigrant or adolescent populations (Rennick-Egglestone et al., 2019; Nickerson et al., 2020; Aarethun et al., 2021), although no work to our knowledge exists in testing the effectiveness of such approaches in African-born immigrants. Specifically, based on our extensive literature review of published empirical or systematic review data on several large Psychology databases with a comprehensive set of search terms (including, but not restricted to, terms such as “African”, “African immigrant”, “African refugee”, “mental health”, “health screening”, “mental health prevention”, and others), the scant data around mental health screening and assessment in Africans has been primarily for those living in Africa (most commonly South Africa; e.g., Duby et al., 2021). However, a few studies have at least examined mental health attitudes and barriers in African refugees (who have typically fled persecution in their own countries) or immigrants in the United States or other Western countries (e.g., Ireland and Germany), where these individuals face additional unique acculturative and social stressors related to post-migration settlement (Gharibian & McCarty-Caplan, 2023; Murphy et al., 2021; Grupp et al., 2019). These three existing studies with African immigrants/refugees utilized qualitative or narrative interview approaches and found very similar findings to our previous mixed methods work with local Utah African communities (Kaur et al., 2022), including denial or lack of awareness of mental health, and stigma towards mental health (Gharibian & McCarty-Caplan, 2023), a preference for religious or medical professionals for treatment of mental health symptoms instead of psychological specialists (Grupp et al., 2019) as well as an interconnection of mental health with other realms of well-being such as physical, emotional, and social health (Murphy et al., 2021).

Recognizing that unique cultural beliefs, values, and broader social determinants of health shape barriers to mental health screening and care utilization among African-born individuals in the U.S., we employed the socioecological model (Bronfenbrenner, 1977; Bronfenbrenner & Morris, 1998) to steer the approach employed in this study. This framework allowed us to identify barriers and facilitators to care uptake at multiple levels (i.e., the individual, interpersonal, community, and societal/policy levels), while allowing the significant incorporation of social structures (e.g., the reliance on family/religious leaders, and the previously stated preference for CHW health service provision) and systematic broader factors (stigma and low mental health literacy) that may influence a particular behavior. Informed by this model, our study aimed to guide the development of community-informed mental health vignettes, based on prior work showing the promise of such an approach in lowering stigma in other forcibly displaced populations (Rennick-Egglestone et al., 2019; Nickerson et al., 2020; Aarethun et al., 2021). We hoped this model would subsequently increase willingness to be screened and to seek care for mental health concerns within this community, as it seems to be a promising approach, given the story-telling cultural norms and desire for a stigma-focused intervention that we observed during our own prior work with this community (e.g., Kaur et al., 2022).

Thus, we co-created vignettes that aimed to provide education on common anxiety-focused symptoms, address stigma, and provide an example of how CHWs can be incorporated into mental health screening and care. In this study, we wanted to subsequently assess the vignettes’ qualitatively reported acceptability (i.e., how appropriate, tolerable, and suitable they are) and utility (i.e., how useful or desirable they are) and these vignettes’ ability to decrease stigma towards mental health (similar to some limited prior work in serious mental illness with other African communities; Digwamaje & Tadi, 2024) while increasing community members’ mental health knowledge and willingness to engage in evidence-based mental health screening. Specifically, we adhered to the community-based participatory research (CBPR) approach (Wallerstein & Duran, 2006) used successfully in our previous work to ensure equal partnership with our community partner to jointly achieve two main study objectives: (1) to engage closely with a community advisory board (CAB) of African community members and stakeholders to co-develop these mental health vignettes to ensure cultural relevance, and then (2) conduct rigorous qualitative data collection and analysis to assess the perceived utility and acceptability of such vignettes with 2 focus groups of community members. Our broader goal was for these mental health vignettes to be included (if deemed adequately acceptable and useful by the community) in a CHW-administered mental health screening protocol designed to support African-born residents of the U.S. It is our hope through describing the methods utilized to develop and evaluate such materials and subsequent findings from our focus groups on these materials that this work could serve as a model for cultural tailoring and responsive development of materials promoting mental health screening to address ongoing inequities in mental health treatment seeking for similar immigrant and refugee groups.

## 2. Materials and Methods

### 2.1. Community Advisory Board

Prior to the start of this study, we created and elicited the guidance from a CAB, consisting of a range of members of the Utah African immigrant and refugee community. Specifically, our CAB was recruited by purposive sampling as led by the Executive Director of our community partner, the Best of Africa Group (author V.M.). It was composed of 5 females and 1 male (including author V.M.), and this composition was intended to capture as wide a range of roles and placements as possible within the community (i.e., community health worker, daycare provider, a student, and community members engaged in other advocacy/service-based work for the community such as assistance on document completion or translation services). Given the pilot nature of this study, we recruited a fairly small-sized CAB (6 members in total with the Executive Director counted as a member) compared to typical CBPR (Wilczek et al., 2025). The research team met with the entire CAB virtually over Zoom (version 6.0, 2024) for three meetings during the summer of 2024 to propose the study idea, which was presented broadly as a plan to develop and assess culturally-responsive vignettes to increase willingness to receive mental health screening and treatment for African-born community members. During these meetings, we presented a planned agenda that included a range of logistics related to the broader study aims to obtain the CAB’s input on all aspects of implementation (e.g., size of focus groups, gender balance, virtual versus in person), the specific focus group questions planned, and the vignettes we hoped to show to the focus groups so that we could collaboratively adapt these to be more culturally appropriate in close partnership with the CAB. The CAB members reviewed vignettes with us in our three 2-hour meetings, suggested features for the stories to make them more accessible, easy to understand, and most relatable/relevant to the community. CAB feedback was integrated into the vignettes before sharing them with the focus group participants. Each CAB member was compensated USD $75 for each meeting.

**Final focus group questions and vignettes.** Following each CAB meeting, the core team (authors S.S., A.A., and V.M.) would meet to go over the suggestions made by the CAB to discuss how best to incorporate their suggestions and develop the questions and feedback to the CAB about how we made changes based on their advice for each subsequent CAB meeting. Through this multilayered, iterative process of regular core team meetings and 3 full CAB meetings, we generated a final list of focus group questions to further evaluate the impact of, and reception to, the co-created mental health vignettes (see Appendix A for facilitator prompts, final focus group questions and presented vignettes).

The two vignettes presented were related stories (part 1 and part 2) about one main character (Jane, as named by the CAB), a mother experiencing emotional and physical symptoms of generalized anxiety disorder (APA, 2022). Part 1 showed Jane describing and experiencing the impacts of her symptoms, and ended on a somewhat hopeless or negative note, and then Part 2 (as advised by the CAB) showed how Jane uses culturally congruent ways to obtain help for her symptoms and come to a place of healing or hopefulness for recovery. The length and language of each story was discussed in close partnership with the CAB, and to align with other vignette/stigma reduction work with other refugee populations (e.g., Rennick-Egglestone et al., 2019).

The focus group questions focused on showing each of these parts of Jane’s story to elicit focus group members’ (1) thoughts, feelings, and emotions hearing the stories presented, (2) how easy each story was to follow, (3) how common they felt the story of Jane was compared to their own families and the community at large (acceptability), (4) how they felt the main character managed her difficulties, (5) how effective these stories might be in encouraging others in the community to seek help (utility), and (6) any elements missing from the stories or general recommendations the focus groups had for the research team to make these stories more effective or broader comments about addressing mental health in the African community.

### 2.2. Community Focus Groups

Author V.M. spearheaded recruitment using purposive sampling techniques to form two mixed-gender focus groups (*n* = 18) with the African community in Utah, which were held in September and October 2024. In line with our previous work (Asnaani et al., 2022), guidelines for focus group methodology (Krueger & Casey, 2014), and stated community preferences, each focus group consisted of 8–10 adults aged 18 years and up, and in consultation with our CAB, we determined that two groups of this size would be most appropriate and feasible for this study. Thus, given the pilot nature of this study, CBPR-related considerations dictated our final *n* rather than a formal saturation test of the qualitative dataset. Participants’ countries of origin included Liberia (*n* = 7), Rwanda (*n* = 4), Somalia (*n* = 3), Kenya (*n* = 1), Nigeria (*n* = 1), Burundi (*n* = 1), and Democratic Republic of Congo (*n* = 1). There were 13 females and five males across the two groups. Interested participants were identified based on their representation of different segments of the community (e.g., students, parents, religious diversity, gender diversity, older adults, etc.) and engagement in community events and initiatives. All participants reported being employed, with most participants reporting working in manufacturing jobs.

**Procedure.** Each potential participant was approached in person or by phone/WhatsApp by our community partner and was provided with an informed consent cover sheet explaining the focus group purpose, i.e., “to examine the usefulness of written stories in assisting the African immigrant community in learning more about common mental or emotional health symptoms and treatment options for emotional health.” If potential participants showed interest in participating, author V.M. provided one of two focus group dates to determine availability and placed participants in each focus group to balance representation across various demographics (gender, age, and religion) wherever possible. Enrolled focus group participants then attended a 2-h focus group that was held virtually over Zoom (as recommended by the CAB to maximize accessibility and attendance) and were compensated USD $100 for attendance.

All study procedures were reviewed and approved by the University of Utah’s Institutional Review Board.

### 2.3. Reflexivity and Positionality Statement

To ensure interdisciplinary perspective in understanding mental health screening and care utilization among African-born individuals in the US, the research team included three female doctoral-level faculty members: two of the principal investigators (PIs), one from the Department of Psychology and one from the College of Nursing, both long-standing collaborators with the Best of Africa community-based organization for more than 15 years, and a key collaborator from the College of Nursing, who provided instructions and oversight throughout the analysis process (who has also supported community-engaged research with Best of Africa for more than five years). The research team also included three junior members: one undergraduate (female) and two post-baccalaureate research assistants (1 male and 1 female) who worked as the core coding team. The third PI was the community partner (author V.M.) who is the Executive Director of Best of Africa, a community health worker serving large segments of the community herself, and African refugee from Rwanda. The research team comprised one West Indian member of South Asian descent (author A.A.) and five White members from both U.S. and non-U.S. (author T.L.) cultural backgrounds.

While two team members identified as immigrants and possessed first-hand understanding of acculturative stress, they also recognized that their experiences might not be comparable to those of refugee participants who experienced forced relocation. Additionally, given the team’s largely non-African composition, we recognized our positionality as outsiders relative to the African-born immigrant and refugee community and the potential for our Western cultural perspectives to influence data interpretation. To minimize these biases and to ensure accurate, culturally grounded interpretation of qualitative analysis findings, the team relied on the expertise of senior team members who have been working with this community for many years and on the insider perspectives of the community partner PI, who played a key role in guiding the team’s efforts and mitigating outsider bias.

### 2.4. Data Analysis

We employed a qualitative descriptive approach (Sandelowski, 2000, 2010), using thematic analysis (Braun & Clarke, 2006) with deductive and inductive coding. This hybrid approach has been used elsewhere (e.g., Taylor-Swanson et al., 2024; Dauria et al., 2021) and allowed us to maintain focus on practical goals (i.e., assessing the vignettes’ clarity, cultural relevance, and effectiveness/impact) while also exploring cultural and social factors unique to the African immigrant communities in the U.S. that shape their understanding of, and attitudes toward, mental health. Further, we followed the Consolidated Criteria for Reporting Qualitative Research (COREQ; Tong et al., 2007) guidelines for the standard implementation and reporting of the qualitative work reported here. Trustworthiness was addressed through multiple strategies (Lincoln & Guba, 1985). Specifically, credibility was supported through collaboration with the CAB in co-determining the study topic and co-developing study materials and procedures. Confirmability was enhanced through review of qualitative findings by the community partner PI (author V.M.) who provided crucial community-based insights that informed the final themes. Dependability was supported through the maintenance of a detailed memo log by the lead research assistant during each team analysis meeting. Analytic memos also included reflexive notes about interpretive decisions.

All focus groups and interviews were audio-recorded and transcribed using Zoom. Raw transcripts were reviewed by the core coding team to ensure accuracy. De-identified transcripts were coded using Dedoose software (version 10.0.25, SocioCultural Research Consultants, 2024). Given that the focus group prompts were structured to capture participants’ reactions to two written stories, we used the focus group guide to develop the initial codebook (deductive coding) and modified it as needed to incorporate any new emerging themes (inductive coding).

To improve consistency and reliability and ensure shared understanding of code application, the first transcript was coded collaboratively by the entire team. After the codebook was finalized, definitions for all codes were agreed upon, and team consensus about code application was achieved, the primary coding team (consisting of 3 coders) had two coders double-code the remaining transcript independently, followed by an audit by the third senior coder (author T.L.) to adjudicate on any disagreements in coding. Finally, consensus meetings with the entire analysis team were held weekly to review coding and resolve any remaining disagreements. Following initial coding, codes were divided between four coders (the 3 initial coders and the PI of the study) who independently reviewed the content of these codes to ensure thoroughness of inductive coding. Ideas that featured prominently were tagged as sub-codes within each of these broad codes. During sub-coding, the four coders also organized sub-codes under four levels of the socioecological model (Bronfenbrenner, 1977; McLeroy et al., 1988; Bronfenbrenner & Morris, 1998): the intrapersonal or individual, interpersonal (e.g., spouse, parent, friend), community, and societal/policy levels, based on whether the quotes relating each subcode aligned with the four specific levels of the socioecological model. That is, tagging of specific socioecological model levels occurred depending on whether the quotes referred to just individuals (their experiences and individual circumstances), their relationship with others, the role of the local community (especially in terms of cultural norms), or if the issue was more global and would not be able to be addressed on a simply localized level. This allowed the team to interpret the findings through a socioecological lens, highlighting multilevel influences on mental health and care uptake within this community (see final column of Table 1). If a sub-code had different quotes that were relevant to multiple socioecological model levels, then that sub-code was tagged with all applicable levels of the model for most accuracy/completion. The team then discussed the results, reaching consensus on the main themes, and selecting representative quotes from the transcripts (see Supplemental Table S1). For the narrative write-up, the findings for each theme were organized by level of the socioecological model.

## 3. Results

The main themes our team reached consensus on surrounding participants’ reactions to the presented vignettes and their perspectives on mental health screening and help-seeking more broadly were: the dual nature of the role of family, community voices as education for addressing mental health stigma, African culture as both a barrier and a bridge, and the importance of stories about lived experience, with specific subcodes mapping on to each of these broader themes (see Table 1). We discuss each of these in detail next.

**Theme** **1.** *Between Support and Strain: The Role of Family.*

Focus group participants expressed family playing a crucial role in mental health outcomes, with some believing that family should be a primary resource for care over providers. For example, when our stories described an individual who benefited from professional mental healthcare, Participant 13 (female) stated, “I feel like she should have talked to her husband more and her kids, and tried to figure out a solution. And then, if they don’t have the solution, go to the counselor, second,” showcasing the shared perspective that individuals should prioritize supportive interpersonal relationships over professional healthcare when in need.

At the same time, there were participants who praised our character, Jane, for getting the help she needed. When it was thought that “Jane’s” problems were beyond what her family could relieve, Participant 2 (female) said that, “I’m glad that she got help that she needed and I like that she talked with her family about it…She was not ashamed to go to the hospital or to go to the doctor.” When speaking of family, there was consensus that, while family was seen as beneficial by some at the individual and interpersonal level, the majority of participants also felt that family could be a detriment to their mental health, often citing it as a potential burden. For instance, Participant 14 (female) explained, “So some moms feel like, ‘Okay, I can’t even go to my family, let me seek help from outside.’ So, I would have done the same … if those were kind of family that I had. They’re busy. They’re not thinking of me and stuff like that.”

When exploring the negative impact of a lack of support from family members, the role of motherhood at the individual level emerged as a key point of discussion. Participants described a wide range of responsibilities that mothers carry and the adverse effects of such gender-specific stressors. Participant 16 (female) shared that, “The story kind of made me feel a bit frustrated. Because I know for me personally, especially in most African households, the mom is expected to [do] everything, since they have more of a traditional mindset…She has her whole family depending on her, and typically she’s not expected to take a rest until she’s able to fulfill her duties as a mother.” Tasks that were listed as belonging to mothers included household cleaning, child rearing, and providing meals for their families in addition to their employment. Many described the isolation that mothers experience, with Participant 1 (female) explaining that “they have to take care of the kids and they put themselves on…as the last option. And that really does something to their mental health. Also want to add that if you need help, ask for, ask for help.” further illustrating compounding factors that negatively affect their mental health at the individual level. When families do not provide understanding or support, the negative impact on mothers’ mental health at the individual and interpersonal levels are further compounded.

Participants felt that, regarding mental health, African norms at the community level trickled down to the interpersonal and individual levels. When speaking of a lack of recognition of mental health at the community level, Participant 12 (female) shared, “It’s not something as African culture. It’s not something we are allowed to do… opening to the people to tell them like the way we’re feeling.” Participants discussed this perceived lack of mental health literacy, many felt that it was present in their own homes at the interpersonal level, with Participant 1 (female) sharing “…if their family don’t like believe in mental health, and they don’t think that’s like something can affect them in real life. Then even though…she, the person, wants help, she will feel like nobody will support her in any way.” There was consensus that this lack of understanding led to mothers feeling burdened and isolated from their own families with their needs being ignored. When discussing our scenario, Participant 2 (female) commented, “She’s thinking I’m doing this by myself. Nobody is helping me, and she’s resenting, resenting her family like her husband.” Within our discussions, there were some who felt that there’s a lack of “teamwork” in their familial relationships at the interpersonal level of the SEM. However, in response to this talking point, there were participants who believed that family could act as a mechanism to seek mental healthcare, with Participant 1 (female) sharing, “It seemed like her family helped her to get through. I feel like if your family or friends who are there for you, [it] will be easier for you to go get help.”

**Theme** **2.** *Raising Awareness, Reducing Stigma: Community Voices as Education.*

Within the *Raising Awareness, Reducing Stigma: Community Voices as Education* theme, participants highlighted a critical need to improve mental health literacy and address related stigma and misconceptions that pose significant barriers to help-seeking among community members. At the individual level, many participants described limited understanding of mental health conditions, symptoms, or coping strategies. For instance, Participant 8 (male) noted, “We are from Africa; I never knew what they call ‘mental health’ until I came back to the US.” Another participant added that very few people in their community “are capable to detect … signs … related to emotional and mental health” (Participant 6, male). This lack of awareness often leads to difficulties recognizing symptoms (i.e., stress, burnout, depression) delaying timely help-seeking. Moreover, many participants agreed that “not a lot of people [in the community] know there’s help out there” (Participant 1, female). Participant 6 further noted that many “don’t have capability and capacity to access [care],” emphasizing the need to engage and train community advocates or health workers to help people connect to the appropriate mental healthcare resources.

Stigma surrounding mental health and a lack of trust toward mental health providers, as found in the current data, present major barriers across all socio-ecological levels. Multiple participants emphasized the importance of creating safe community spaces that provide support and information about available resources without judgment. Particularly, several participants highlighted the value of hearing personal testimonials from other community members to negate misinformation about mental health, reduce shame, and normalize help-seeking. For example, Participant 9 (male) explained, “a lot of time, people open up when they hear other people testimony. So, if you have the experience in telling someone what you went through, become more vulnerable … your testimony can be able to help them to come out and then to seek the necessary help and resources that they need.”

Participants also shared ideas on addressing mental health stigma and knowledge gaps to facilitate early recognition and treatment of mental health issues among African immigrants. They emphasized the need to have easily accessible and culturally relevant resources and care tailored to diverse populations and languages. At the community level, most participants agreed on the importance of engaging community leaders and trusted providers from refugee and African backgrounds in outreach activities and to raise public awareness. Specific recommendations included offering free consultations and screenings, holding educational seminars and workshops, creating support groups, sharing stories that reflect community experiences, and using social media campaigns and flyers. At the broader societal level, participants recommended policy efforts and funding to support culturally responsive, inclusive outreach and education programs.

**Theme** **3.** *Culture as a Barrier and a Bridge.*

Focus group participants expressed that African immigrants often struggle to seek mental healthcare due to traditional African attitudes that discourage treatment. When prompted, participants spoke of the stigma that exists in the community and how it affects their willingness to acknowledge and share their own mental health struggles with others at the individual and interpersonal levels. As Participant 12 (female) expressed, “…as African culture, it’s not something we are allowed to opening to the people to tell them like the way we’re feeling. But these days I think it’s something we need to do, because there’s a lot of things going on.” This stigma, in combination with a lack of mental health literacy, leads to dissuasion from participation within the mental healthcare system at the individual level. Participant 10 (female) stated that, “Because in our African community, when we say we are depressed, sometimes I’ll be like, ‘Oh, your stomach is too full. That’s why you depressed.’ They’d be like, ‘oh, you have house, you have all these things, they say. Why are you so ungrateful? You have all those things you don’t need to be depressed’ was kind of like really affecting us, because sometimes, when you’re so depressed, our family don’t look into that.” Differences in perception of mental health between genders were also raised. For instance, Participant 11 (male) believed that men and women’s behaviors were distinct from each other at the interpersonal level, mentioning, “I think men are more hesitant to go out to seek mental health issue because…They want to have the ego. They don’t want to see to be seemed as to be seen as weak. Because for men speaking about what’s going in their lives, it is the sign of weakness.” Such stigmas as the ones listed above lead to feelings of isolation from the individual, interpersonal, and community levels.

However, even when mental healthcare is explored as a treatment option, many African immigrants face barriers from providers who lack knowledge specific to their cultural background. Many participants mentioned that non-African mental healthcare workers are unequipped to help them, expressing wariness about the effectiveness of treatment from an individual with no knowledge of the culture they come from. As Participant 8 (male) explained, “If you…take a white person and taking their community to explain, you know, to our people about mental health, they won’t get it. If they give you how to survive mental health, wouldn’t, because my African way of mental health is different than they are so open.” Participant 1 (female) brought up that community members face more obstacles from accessing mental healthcare due to language barriers, “…I know a lot of people who they don’t speak the English language. If we can get people who can translate everything and where they understand where they can get help and who they can call and all that stuff.” Suggestions for improvement include providers familiarizing themselves with African culture and participating in cultural events, free screenings, collecting testimonials from within the community, creating safe spaces for discussions about mental health, and creating incentives to encourage community members to participate in such spaces. However, as it stands, most participants share hesitations that they can be helped by a system that doesn’t understand the culture of African immigrants.

Additionally, a consensus across focus groups was advocating for improvement at the community level. In our discussion, many participants felt that members should be able to speak more freely about mental health at the community level and normalize such topics. Participant 2 (female) stated that, “In general things, even in Africa things should change. this is very, very common in our African community…So, I think this like within this, our community, I think people should start to be aware of the way they can ask for help.” This shared belief sparked conversations about changes that can be made at the community level. Spreading awareness, community outreach, and educational seminars were all suggestions that were proposed. Some participants spoke of changes that they are already seeing in their community, specifically younger generations being more open to the idea of mental healthcare, with Participant 16 (female) sharing, “I realized that for the younger generation we’re really open to mental health. Because we kind of have social media bit of a younger generation. But I feel like the community that really needs to be targeted are the elders, because for them mental health is pretty much non-existent.”

**Theme** **4.** *Stories that Reflect Lived Experience.*

Participants shared their feedback about the written vignettes and their impact. On a personal level, many agreed that the stories felt real and easy to relate to, especially when it comes to the family dynamics, women’s experiences, and the multiple expectations placed on mothers. For instance, Participant 11 (male) noted, “It’s like a scenario that have been picked from a day-to-day experience of an African family, so almost everybody can see themselves in that scene.” Participant 8 (male) shared another common sentiment, explaining that it’s very typical for “African moms [do] everything at home … to clean the house, take care of the kid, cook, … everything, while she have her own extra job outside.” This emphasized gendered division of labor and responsibilities placed on women at the interpersonal and community levels. Participant 2 (female) added that in the African community, women are often “scared or afraid to ask for the help,” underscoring how cultural norms and expectations can contribute to stigma and discourage open conversations about emotional struggles, further limiting help-seeking behaviors.

Many participants agreed that shame and fear of being judged rooted in mental health stigma remain major barriers across all socioecological levels. With that in mind, many participants appreciated the positive tone of the stories and the character’s willingness and courage to seek help, despite the stigma. Participant 16 (female) explained, “I’m kind of glad that she got the help that she needed, and … that she was brave enough to go, [even though] she was afraid of what her community would say. … That’s really prominent in African communities, since there’s a lot of stigma around mental health, and most people don’t really acknowledge that it’s real.” Many other participants also found it powerful and motivational to see someone reach out for help despite cultural pressures, “It was really encouraging to hear all of it” (Participant 14, female).

Some participants wished that the written stories included more specificity on real-life barriers and offered concrete guidance. For instance, Participant 16 (female) suggested including more practical details, “about how [the character] found the counselor, the steps she took, [things] she had to do on her own. It would probably encourage more people.” Another participant expressed confusion about whether the story was about Africans in general or African Americans, noting the importance of recognizing differences in cultural upbringing (Participant 11, male). Other recommendations for improving such stories or screening intervention more broadly involve simplifying language, offering non-English versions, and ensuring that narratives reflect a diverse range of identities, including gender, age, and religion, to meet interpersonal and cultural needs.

## 4. Discussion

In this study, we examined community responses to community-developed screening vignettes that described a member of the African community experiencing common mental health symptoms (chronic anxiety) and her efforts to receive help for these issues. Specifically, we worked with a CAB to develop elements of these stories, and we then examined focus group participants’ perceived utility and acceptability of the stories. Responses to our prompts in these focus groups were categorized using levels of the socioecological framework to illustrate the various levels of influence (individual, interpersonal, community, and societal/policy level) that play a role in shaping mental health perceptions and treatment-seeking behaviors (Sharma et al., 2020). The mapping process revealed multiple areas of overlap between different sub-codes and levels, highlighting the complexity and interconnectedness between these factors in influencing mental health outcomes. To capture these broader influences, we organized our findings into four overarching themes, which were (1) between support and strain: the role of family; (2) reducing stigma: community voices as education; (3) culture as a barrier and a bridge; and (4) the importance of stories that reflect lived experience. In general, we observed that family dynamics and mental health literacy gaps were most impactful at the individual and interpersonal levels, while issues such as access to mental healthcare education and resources and lack of culturally competent providers were most prominent at the community and societal levels. Stigma, gendered norms, and cultural identity factors cut across every theme and level, underscoring their significance. Overall, participants showed a high degree of identification with these culturally responsive vignettes and provided confidence in our ability to utilize these stories as part of a future screening intervention for this community that aims to target stigma reduction and increased help-seeking.

### 4.1. Implications

Overall, this community-partnered study raises several important implications for future development of mental health promotion and mental health screening materials for African and other immigrant communities. First, the study findings highlighted the ongoing high level of stigma towards mental health in these communities, as has been noted in other studies within African communities more broadly in Western contexts and within their countries of origin (Yılmaz et al., 2025; Ambrose et al., 2025). Given the scarcity of research specifically with African-born individuals living in a Western context, this was important to replicate and adds to the literature identifying stigma as a major barrier to screening and treatment efforts in resettlement contexts. Second, a consistent theme across the CAB meetings and focus groups was around the power of having stories of lived experiences from community members to destigmatize mental health problems, which is a feature of stigma interventions that have been occasionally used with other African groups and health areas (e.g., frequently in HIV work within African countries; Boakye & Adjorlolo, 2025), but the utility of which has never been discussed in such a direct way to specifically target common mental health symptoms with African immigrant communities.

As we remain committed to addressing the ongoing disparities related to detection and treatment of mental health disorders for African-born residents of the U.S, understanding the features that may target stigma and encourage willingness for community members to engage in screening procedures becomes paramount. Our qualitative findings here highlighted such potential features, including narrative stories of community members living with, and importantly, addressing their mental health symptoms using a mix of community-congruent and evidence-based approaches. Third, our findings also provided us with rich data around how community members responded to vignettes that include both culturally-congruent elements and diagnostic symptoms of common anxiety issues, with a generally positive response to seeing the protagonist (Jane) in these stories experience and respond to her mental health symptoms. Interestingly, there was an expressed desire from community members (and the CAB) for even more exposure and dialogue around mental health using public health campaigns, support groups, and printed literature/social media infographics, and testimonials of those who have experienced mental health challenges to effectively destigmatize mental health struggles, all of which we will plan to incorporate in the design of our future screening intervention study.

Taking an even broader perspective than what we specifically found to augment mental health screening efforts in this qualitative study, the study procedures herein continue to highlight the distinct advantages of conducting strong community-engaged work that brings community voices to the table at the inception of a study through dissemination and implementation of its findings, in line with good community-based participatory research principles (Wallerstein & Duran, 2006). Indeed, having a strong community partner such as the Best of Africa organization and their leadership (author V.M.), forming and engaging a CAB, utilizing focus groups to examine and shape screening-promotion materials such as the vignettes explored here, and ensuring a wide range of community members can weigh into our onward screening and intervention efforts provide the greatest chance of making this work sustainable and impactful, versus traditional methods where researchers and community participants are not able to interface in this way (Asnaani, 2023). We have strong evidence to support the idea that African-born communities face considerable barriers in access to healthcare in the countries they have resettled in, and ensuring these communities are themselves part of generating the solutions with such work is crucial for us to adequately address these disparities and increase the overall societal benefit.

### 4.2. Limitations

Despite its strengths and novel approach to the African immigrant community, this study operated within certain notable constraints. For instance, it would have been ideal to obtain data from several more focus groups, and to check in with the CAB between focus group cohorts (which we also would have liked to be larger and even more representative in terms of occupation, gender, and age than the 6-member team we assembled for this study), but due to funding constraints, neither additional focus groups or an expanded CAB were possible. Relatedly, funding options for such work have been considerably limited since (and shortly after) the data collection for this study was completed due to the current sociopolitical climate in the United States that has deprioritized the care and services for refugee communities in particular. Specifically, the forcibly displaced segment of the African community in particular is currently experiencing significantly decreased availability of social services due to federal funding cuts (Rfat et al., 2025), and we need to examine how this may be impacting (1) the broader community’s current mental health needs and (2) community and individual priorities given the current sociopolitical climate. Particularly given our reliance on the socioecological model (Bronfenbrenner & Morris, 1998) to understand the current study findings, future work conducted around screening interventions would benefit from explicitly and systematically incorporating information and responses from participants on how such current sociopolitical events may impact various levels of this model, which we were not able to do here given the timing of data collection.

Other limitations of the study include our unique population of African immigrants and refugees living in a predominantly non-Hispanic White population in Utah and the geographical roots of our participants (who hailed specifically from the countries of Liberia, Rwanda, Somalia, Kenya, Nigeria, Burundi, and Democratic Republic of Congo), who may have different experiences and cultural views from those who come from different regions of Africa. Similarly, all of our participants were either adult first-generation immigrants or refugees, and we know from our prior work with this community (Kaur et al., 2022) that there is a large divide between the first and second generation (or first-generation children who were quite young when they came here) in terms of mental health needs and stigma toward mental health; therefore, future work would benefit from understanding how we can use developmentally appropriate vignettes to resonate most effectively with different age groups at various stages of acculturation in the community. Similarly, we had a majority of women in our sample, and therefore many of the themes revolved around women’s experience of mental health in the community (and the main protagonist in the narrative stories was also female). It would be helpful to recruit more males given the stigma around mental health and its association to “weakness” particularly for men in the community in future work as well as to develop future vignettes that focus on a male character’s experiences with mental health.

### 4.3. Future Directions

Nevertheless, this study’s findings provide a window into how we can culturally tailor approaches to destigmatizing mental health in the hope that we can promote the uptake of screening for African-born communities. Improvement of mental health screening is a crucial first step to ensuring adequate utilization of mental health services (and utilization of other community-preferred ways of help-seeking, including community support), so that we may appreciably address ongoing health disparities and subsequent trickle-down effects on the overall health, well-being, and productivity of all members of our society. Our team is currently testing an application of these vignettes within the context of culturally-informed psychoeducation and psychometrically valid measures for common mental health symptoms (with all components delivered by trusted CHWs from the African community) compared to routine psychoeducation and similar measures without presentation of these vignettes (delivered by trained Psychology and Nursing professionals). The study employs a randomized group design to test whether addition of such brief narratives prior to offered screening by CHWs can directly lower reported stigma towards mental health, improve health-related knowledge/literacy, and most saliently, encourage community members to engage in screening to appreciably improve screening rates for the community and subsequently, greater mental health service utilization over a 3-month follow-up period for those community members who need them. The current study and future/ongoing study we are engaging in as the next step of this work provides support for a low-cost but potentially effective avenue in which to encourage screening and, ultimately, treatment-seeking for those members of this community who need it most, and this could serve as a helpful model for the large number of immigrant communities in the U.S. overall.

## 5. Conclusions

This study explored African community members’ perceptions on the acceptability and utility of vignettes of a representative African community member experiencing common mental health concerns (specifically anxiety symptoms) and how this character navigates receiving treatment within the currently existing social structures of the United States to achieve symptom relief and improved quality of life. The study employed a CBPR approach to ensure equal partnership and co-development of materials and study procedures with community members from the large African immigrant/refugee community in the Mountain West region of the United States, finding overarching themes from the qualitative data collected, namely the dual role of family in being sources of support and strain, the importance of using community voices to reduce stigma and increase health education, the double-edged nature of African culture as a barrier and bridge to wellness, and the high value placed by this community on stories of lived experience to encourage positive health-related behaviors, including willingness to be screened and treated for common mental health concerns. The vignettes assessed here are currently being incorporated into a culturally-responsive screening protocol that is delivered by African CHWs in conjunction with culturally-relevant psychoeducation and standardized, translated screening measures for common mental health disorder symptoms to test its comparative effectiveness compared to standardized screening by medical/psychological professionals in improving screening rates and subsequent treatment utilization. It is hoped that the current study has elucidated the needs of the African-born community in the United States and provided a blueprint for the creation of additional materials that incorporate story-telling community preferences to improve mental health screening and treatment-seeking to more appreciably address ongoing mental health inequities in this community.

## Figures and Tables

**Table 1:** Overarching themes, sub-codes and socioecological model (SEM) levels for data.

Overarching Themes and Sub-Codes	SEM Level(s)
**Between Support and Strain: The Role of Family**	
Family support/lack of support; seeking or not seeking help	Individual, Interpersonal
Lack of family recognition of symptoms	Individual, Interpersonal
Family involvement/responsibility in mental health	Interpersonal
Gendered family roles, expectations, and responsibilities (esp. mothers)	Individual, Interpersonal, Community
Tradition vs. assimilation in childrearing	Interpersonal, Community, Societal
**Raising Awareness, Reducing Stigma: Community Voices as Education**	
Stigma, mistrust toward providers	Individual, Community
Mental health literacy gaps	Individual, Interpersonal
Lack of understanding/recognition of mental-health symptoms	Individual
Lack of coping skills and strategies	Individual
Lack of knowledge about available resources for help	Individual, Community
Lack of resources for and education about mental health, lack of public awareness	Community, Societal
Limited culturally tailored services	Community, Societal
Recommended interventions: Trusted testimonials, free screenings, flyers, social media, educational seminars, incentives	Community, Societal
Recommended interventions: Policy level/educating systems of care on African culture, free resources, inclusive outreach, incentives to engage community members; funding	Community, Societal
Need to have safe space/trusted people within community	Individual, Interpersonal, Community
**Culture as a Barrier and a Bridge**	
Cultural gender roles, gendered stigma and/or discrimination (women’s burden, cultural norms)	All levels
Mental health beliefs and misconceptions; stigma, self-stigma	Individual, Interpersonal, Community
Cultural and generational differences in perception of mental health	Individual, Community
Acculturation stress; language barriers	Individual, Interpersonal, Community, Societal
Mistrust of non-African providers	Individual, Interpersonal
Symptom invalidation in community; lack of support/understanding from the community	Interpersonal, Community, Societal
Community stigma, rejection, ostracization	Interpersonal, Community, Societal
Need for culturally sensitive/trusted providers (incl. non-English services)	Community, Societal
**Stories that Reflect Lived Experience**	
Relatability of Jane’s struggles (family dynamics, gender differences, lack of support, stigma)	Individual, Interpersonal, Community
Improving stories: More details about finding help (how/where), more details about treatment process and what happens after	Individual, Interpersonal
Tailoring recommendations: Narrative stories with more representation	Individual, Community, Societal
Tailoring to cultural identity, gender, age, religious beliefs, language options	Individual, Community, Societal

## Data Availability

Non-identifiable qualitative representative quotes are provided in the supplement. Given the qualitative nature of the data, we cannot provide raw data (i.e., focus group transcripts).

## Supplementary Materials

The following supporting information can be downloaded at: https://www.mdpi.com/article/10.3390/bs16060993/s1.

## Appendix A

**A. Facilitator:**

- *“We are meeting today to provide our perspectives and opinions on how written stories may help our community feel more comfortable with discussing mental health and getting screened for mental health problems.*
- *We hope that by sharing with each other today, we can help our community get more connected to the emotional health services that exist. There is no wrong answer, and anything you want to share for each of the questions we ask is just fine. You can share as much or as little as you like, but we would love to hear from all of you.*
- *We will talk for about two hours today, and we will audio and video record the session so we can make sure we don’t miss anything. We also want to keep everything we discuss today as confidential in our group, so please use headphones or find a private place for us to chat if possible.*
- *After today’s session, we will pay each of you a $100 Amazon gift card for your time spent with us. Before we end today, we have an email address for each of you to send that information. Any questions?”.*

**B. Facilitator:** *“First, we will look at a story about an African woman and then ask you some questions about your reactions to her story afterwards.”*

**Revised Written Story #1:** [FACILITATOR READS OUT LOUD AND STORY ALSO SHOWN IN WRITING TO PARTICIPANTS].

Jane is working in her kitchen at home, making rice for the family. She has just returned from work and her husband and children are also coming home from work/school. As she mixes her rice and onions for the pilau she is making, she starts to think about all the chores she needs to do tonight and notices her neck and back beginning to ache. Her heart starts to race and she tries to concentrate on her cooking but finds it hard to stop thinking about everything she needs to do. Her 16-year old son Charles comes into the kitchen and looks over her shoulder and says, “Mama, I’m hungry!” and grabs a bite of the food. Jane starts getting mad at him for doing this, even though normally she would not be bothered by it. She just wants dinner to be over so she can rest. Now she is starting to have another headache, which has been happening a lot lately. Also, she has been having so much trouble falling asleep over the last few months that she doesn’t know if she can even look forward to bedtime. She just wishes she could feel calm and feel like herself again, instead of so nervous and overwhelmed all the time.

**Facilitator:**

- *How did this story make you feel?* [If it doesn’t come up naturally, ask them what emotions, thoughts, and physical feelings they noticed while listening to this story].
- *Was this story easy to follow?*
- *How common do you think Jane’s experience is in your family and your community?*
- *Are there any other emotions, physical feelings or thoughts that are common when someone is anxious or worried that were missing in this story?*

**C. Facilitator:** *“Thank you for your input on that first story. Now, we will look at a second story about Jane and what happens after, and then ask you some questions about your reactions to this second part of her story afterwards.”.*

**Revised Written Story #2:** [FACILITATOR READS OUT LOUD AND STORY ALSO SHOWN IN WRITING TO PARTICIPANTS].

Jane is tired of feeling so nervous and worried all the time. She decides to share her feelings with her husband and oldest daughter to see what they think she should do to feel better. Her daughter Sifa tells her about a clinic she heard about from her youth group at church that specifically works with people who are feeling stressed or nervous. Jane is a little scared to go such a place in case they don’t understand her or what she is feeling. She is also worried about what others in her community would think if she went to such a clinic. Her husband gently encourages her to at least talk to Erica, who is a community health worker and also a friend from the same community, to see what she thinks. Erica listens to some of the things Jane has been struggling with, and encourages her to go and see what the people at this clinic have to say. Erica helps Jane schedule an appointment at this clinic. She also goes with her for the first visit. Jane is very nervous when she goes to the appointment because she is not sure what it will be like. But the counselor meeting with her is very welcoming and asks her questions she can answer about herself. She meets with this counselor for about an hour, and then at the end they discuss some options to help her with her problems. These include learning new skills to manage her stress, talking about what is stressful in her life, and medications as another option. Erica explains these options to help Jane make a decision that she is most comfortable with. Jane feels hopeful about the treatment option she has chosen, and that she doesn’t have to struggle with this stress forever.

**Facilitator:**

- *How did this story make you feel?* [If it doesn’t come up naturally, ask them what emotions, thoughts, and physical feelings they noticed while listening to this story].
- *Was this story easy to follow?*
- *What do you think about how Jane handled her difficult experiences?*
  - *Were there things she did that you would the same as her?*
  - *Were there things that you would do differently from what she did?*
- *Do you think Jane’s story would encourage someone to seek help for similar problems? Why or why not?*

**D. Facilitator:** *“Thank you for that additional input. As we come to the end, we have a few last questions for the group.”*

- *What recommendations do you have about showing these stories to people in the community to encourage them to get screened for mental health problems?*
- *Any other general recommendations on this topic after this focus group?*
