# Supplementary material for: Taking a Community-Partnered Approach to Developing Culturally-Responsive Mental Health Screening Materials for African-Born Adults in the United States
Source: Behav Sci (Basel). 2026 Jun 15;16(6):993. doi: 10.3390/bs16060993 (PMC13295341; doi:10.3390/bs16060993)
Supplement: Supplementary file 1 [file behavsci-16-00993-s001.zip › BoA Qual findings supplement FINAL 4.14.26.pdf]

Taking a Community-Partnered Approach to Developing Culturally-Responsive Mental Health Screening Materials for African Refugees in the United States

SUPPLEMENT

Supplemental Table S1: Codes, subcodes, and quotes extracted from data.

Code	Definition	Sub themes	Examples
**I. Impact of written stories**	Participant comments about how the story made them feel; call for action	1. Gendered expectations on mothers	**P16 (female):** The story kind of made me feel a bit frustrated because I know for me personally, especially in most African households, the mom is expected to [do] everything, since they have more of a traditional mindset. So, it doesn't matter whether she's working or she has school whatever. Whenever she comes back home, she has her whole family depending on her, and typically she's not expected to take a rest until she's able to fulfill her duties as a mother.

		2. Importance of seeking/having help and support from family members	**P13 (female):** I also think it's nice that she seeked for help. It's a really good thing, and I loved how everybody was helpful when she talked to her family, her daughter, and her husband, how they were helping her figure it out, and like the community. But I also agree with [PARTICIPANT NAME] a little bit, because I feel like she should have talked to her husband and kids more about the situation, because if she goes to a counselor the counselor it's going to be like, 'Oh, can you guys try to like make a schedule of how to like, you know, manage the household stuff,' and things like that. So, I feel like it's good that she went to the counselor for additional like advice and stuff, but I feel like she should have talked to her husband more and her kids and tried to figure out a solution. And then, if they don't have the solution, go to the counselor, second.<br><br>**OPPOSING VIEW**<br><br>**P14 (female)**: So, some moms feel like, 'Okay, I can't even go to my family, let me seek help from outside.' So, I would have done the same situation if those were kind of family that I had. They're busy. They're not thinking of me and stuff like that. That's how, that's how I would have handled the situation.
		3. Emotional impact:	

		3.a. Negative (after 1st story): sadness, frustration (mostly related to gender role expectations)	**P13 (female):** For me, I feel like this story is really sad, but also relatable, because, as everybody said, mothers go through a lot. They're the ones that do like most of the house chores and everything. And it can be … overwhelming sometimes. And I feel like all mothers go through it. So, that's something that like they all share. And I feel like there is a way to avoid it. Avoid it from happening. But sometimes it's hard. Yeah.
		3.b. Positive (after 2nd story): hope, encouragement to seek help	**P16 (female):** For me, it was kind of hopeful as well as happy as one. I'm kind of glad that she got the help that she needed. And it shows that no matter what you're going through, there's always hope. And at the same time, also glad that she was brave enough to go, because in the story she talks about how she was afraid of what her community would say. And I feel like that's something that's really prominent in African communities, since there's a lot of stigma around mental health, and most people don't really acknowledge that it's real. So, if they see you going to a therapist, they think, number one, you're just wasting your money, or you're making issues out of nothing.
**II. Understandability of written stories**	Participant comments about whether the story was easy to follow and understand.	1. Identifying self in stories	**P11 (male):** Yeah, I think I'll just repeat what has been said. The story definitely makes sense, because … it's relatable to most of us. We can see ourselves. Well, you can see yourself in that kind of story. So that makes that makes it really important
		2. Emphasis on simplicity of language	**P13 (female):** It was easy to follow and easy to understand. There wasn't really anything complicated about it.

		3. Language used (English vs other languages)	**P7 (male):** Sometimes there's like a story written in English, but because of difficult grammars used you can't easily understand. But for this one it's really self-explanatory. It's easy to follow.
**III. Degree of relatability of written stories**	Participant comments about how the story compares/relates to their own or other community members' experiences. For example, participant comments about whether they would approach the issue in the same way or in a different way compared to what was presented in the story. Or, other aspects of the story that helped them to relate to the story, such as the woman's racial and cultural identity.	1. Identifying with gendered experiences in written stories	**P1 (female):** I think it's most of the time, we don't realize how many people who they go like through stress like this, especially women who have kids, and they have to take care of the kids. And they put themselves on like as the last option. And that really does something to their mental health. Also want to add that if you need help, ask for, ask for help. And it's really hard to ask for help. I know that as a woman from Africa. nd we're trying. It's... it feels like we're trained to do this, to do the whole thing by ourselves. But we don't have to.
		2. Relating to differences in cultural norms:	
		2.a. Differing child-parent dynamics<br>* Making kids help (kids in Africa are expected to help from young age vs US norms where you can't make children work)	**P12 (female):** ...I think the reason why the mother is kind of stressed. ... I don't know if they can let the kids starting, helping the the parents, because, I'm not sure about sometime when the kids trying to hop in, the parents do something, They say it's like kids abusing like they do something they don't supposed to do. But if the mother came from in Africa and they move over here, in Africa if the kids starting, getting 5 years old, they starting can go get water, they can come, try to helping. The parents do like little things. But when they came over here because of the culture, is kind of different. They try to acting different, and if their parents try to tell them, they say, Oh, no, we have the right to do this, and this. I think it's something we can... I don't know.

			They can, helping us to let the children, our children, to understand. Like where we came from and where we are now.
		2.b. cultural beliefs about mental health:<br>* being raised without education on mental health, thus not knowing much about symptoms or help resources<br>* fear of sharing struggles and seeking help because of stigma	**P6 (male):** We don't have enough knowledge about the problem itself. We don't have knowledge [that] we are to seek for help. Even help may be there. We don't have capability and capacity to access that. So, if I can do any advocacy, I wish we can have more people get educated from the ... refugee background, or African background. Because we don't have this much knowledge back in our countries. When we get here, we face a lot. Then sort out. Be open. Search for help. All these kind of things. It's really hard. So ,I wish we can have more individuals in communities that get much involved to help because we stay there. We know what is going on, we coming from that background.<br><br>**P8 (male):** I never knew what they call mental health until I came back to the U.S., and I kind of ... realized about myself, as well like how things go up with stress. So, for her, from the beginning, she was taught, 'Okay, this normal to feel this way because African setting.' This is what you go through back home. It's our normal life. So, she never recognized it was like a mental health until someone was able to make her recognize, 'Hey, this is not just a normal thing that we go to in Africa. Here is a different

			environment. There is a help for that. This is what you will do, and this is what you can do.' And before you are able to, you know, recognize that, 'Oh, isn't just normal thing that happening to me but rather it was a mental issue,' is something that you know I can see helpful.
		3. Appreciation of positive outcomes within the stories and character's positive traits	**OPPOSING VIEW** (Ways in which this story is not typical in African community)<br><br>**P18 (female):** That story was a kind of a success story. And I like the way everything very simple. So, majority of the time, in our community, we don't have that success story. We don't have someone who actually believes you have a problem. We don't have someone who actually initiates to help you.

**IV. Gender roles**	Any participant discussion about gendered experiences.	1. Gendered distribution of responsibilities in the home (the “second shift”)	**P8 (male):** I think what causes stress for more is that like she’s, she 100% have to be in a home as an African ... mom like who does everything at home. Then as a dad, I only be able to go and take my food and eat, and then go to work. But then my wife there to clean the house, to take care of the kid, to cook. You know, everything. While she have her own extra job outside. So, I think a very relatable in some homes is happening. And that always, always stress, you know, our mothers.
		2. Barriers to good mental health specific to motherhood	**P10 (female):** Being a mother’s kind of like, really hard and stressful. Just stay at home. Keep doing the same thing over and over. Sometimes we need to change our ways, like, if we are depressed. We need to go out and do some stuff for the kid, instead of just being inside the house and doing the same thing over and over again, which is, like, it’s really bad to our mental health. Yeah, changing our pattern of being a parent.
		3. Gender differences in approach to mental health	**P11 (male):** I think men’s approach to mental health is way different to how women approach within our African families. Yes. Think, having a separate groups, because ... men, from my point of view, I think, men a more hesitant to go out to seek mental health issue because they are... They want to have the ego. They don’t want to kind of... They don’t want to ... be seen as weak, because for men speaking about what’s going in their lives, it is the sign of weakness.

**V. Facilitators to good mental health**	Participant suggestions to support mental health among community members (e.g., beliefs, needed education, understanding mental health symptoms, advice/suggestions what might help feeling better, etc.). This is related to what helps them improve their understanding or recognition of mental health symptoms and mental health wellness.	1. Seeking family/friends/ community before counseling	**P2 (female):** I would suggest that JANE ask for help, especially like her son. He is a teenager. She should ask him to help her. That will reduce the stress level. And she should also ask for the husband for help. Because this is, this is very, very common in our African community, and women in general, they are like scared or afraid, afraid to ask for the help. So, I think this like within this... our community, I think people should start [to] be aware of the way they can ask for help.
		2. Need for openness and honesty	**P10 (female):** We should seek help whenever we need it because sometimes we have like hard time in our life, we need to talk to people, to ask people on our mental health.
		3. Cultural Gender norms/expectations	**P6 (male):** I wish one day I can make more community members understand how important it is to open doors and windows for women, girls to be able to relax a little bit.
**VI. Barriers to good mental health**	Participant comments about potential barriers to mental health for community members (e.g., stigma, stress, poverty). This is specifically in reference to what gets in the way around understanding, acknowledging, or recognizing mental health symptoms or the concept of mental health/wellness/illness for the community.	1. Being overwhelmed with responsibilities and expectations (gender specific)	**P6 (male):** much bigger part of these responsibilities goes to women. ... Everyone from Africa knows mothers, women, or girls in charge of this routine - taking care of whole family, do as much as they can to make sure the family is happy. ... Then, when I get here, I find out they're getting even double responsibilities with low income. ... based on my observation, African women and girls [are] still locked in the in the houses, focusing on routine, same thing over and over.
		2. Cultural and generational differences in perception of mental health	**P16 (female):** I was just kind of thinking about this, and I realized that for the younger generation - we’re really open to mental health because we kind of have social media. ... But I feel like the community that really

			needs to be targeted are the elders, because, for them, mental health is pretty much non-existent. Even if they do have it, they don’t acknowledge it, or they don’t even believe it’s a real thing.
		3. Stigma, cultural expectations, and mental health misconceptions	**P11 (male):** I think the story is actually … like a continuum of a story that we … often tell ourselves. Or so the thing that we hear most often in our own families, in our own relatives, right? ... Because they feel like, if they bring that issue, or their concerns in their own family, of course they will be labeled as crazy, or like they have been Americanized, you know, ‘You’re too spoiled.’ So, there’s just that huge stigma that is attached to it that we are... that we are too afraid to tell people ... about these mental health issues.
		4. Limited mental health education	**P1 (female):** As I was saying, it’s not really easy, knowing, at least, from the culture, and you know, and how you think to go get help. I feel like not a lot of people know there’s a help out there.
**VII. Mental healthcare facilitators**	Participant suggestions to support treatment among community members. This is related to what helps community members actually seek help or treatment for their mental health symptoms, which can include talking to someone, getting support from the community, or	1. Family and social support you can trust (testimonials)	**P9 (male):** So, a lot of time people open ups when they hear other people testimony. So, if you have the experience in telling someone what you went through, become more vulnerable, you know, to tell you what they’re going through. ... If you already have like an experience and you have a friend who’s going through that kind of problem ... your testimony can be able to help them to come out and then to seek the necessary helps and resources that they need to also help them.

	formal treatment options.		
		2. Knowledge on available resources for mental health	**P9 (male):** So, all we have to do, first is to acknowledge, and then we have to, you know, communicate, and then the resources, we'll be able to ... get direction where we can get resources.
		3. Logistics of intervention: groups, non-English options, free screening/consultations/educational seminars	**P1 (female):** I think groups. It's a great idea. Also, like, I know a lot of people who they don't speak the English language. If we can get people who can translate everything and where they understand where they can get help and who they can call and all that stuff. Yeah, that would be great.
**VIII. Mental healthcare barriers**	Participant comments about potential barriers to mental healthcare for community members (e.g., access to care, cost of care, stigma within the clinical setting, time, transportation). This is specifically what gets in the way of getting help for their mental health symptoms or seeking formal treatment.	1. Stigma about mental health and meaning of mental health sx	**P10 (female):** In our African community, when we say we are depressed, sometimes I'll be like, 'Oh, your stomach is too full, that's why you depressed.' They'd be like, 'Oh, you have house, you have all these things.' They say, 'Why are you so ungrateful? You have all those things. You don't need to be depressed.' ... Because sometimes, when you're so depressed, our family don't look into that, even the society, they start judging us, you know? They will say all kind of sort of stuff.
		2. Lack of support from family, friends, community	**P12 (female):** In African culture, ... we are not opening to tell the people how we're feeling. Like if I came and I told the people how my family is going on, they're gonna laugh at me. It's not something as African culture, it's not something we are allowed to opening to the people to tell them like the way we're feeling. But these days I think it's something we need to do, because there's a lot of things going on.

		3. Lack of education on common mental health symptoms (not recognizing symptoms) and available resources	**P6 (male):** So as a refugee or immigrant, ... we don't have enough knowledge about the problem itself. We don't have knowledge we are to seek for help. Even if help may be there, we don't have capability and capacity to access that.
**IX. Missing elements from written stories**	Participant comments about common aspects of mental health that were missing from the written stories. For example, participant reported emotions, physical feelings or thoughts that are common when someone is anxious or worried that were missing in the story.	1. Missing specificity of how Jane got help in the story and after the story ends	**P18 (female):** So, I wish if you could get like down the line, ... what happens to JANE. ... Yeah, like it shouldn't end there. Like she just go one session or two. No, it should be like a long-term thing.
		2. Family involvement/responsibility in mental health	**P2 (female):** She's thinking, 'I'm doing this by myself. Nobody is helping me.'And she's resenting her family. Like, her husband, her kids, cause their kids are grown up. So, I would suggest that she asked for help, and that will help her with all the issues that she's having.
		3. Missing inclusivity in the sense of different family members, gender, and culture in the story	**P14 (female):** When it comes to our like, our dads, our brothers, you know, not just mother. Including everybody, because there's some people who are going through a lot, who are like, it can be a lot of people that go through those kind of situation as kids, as all of that. So, it will be good to have this kind of stories to ... involve ... every family member, every brother or sisters, everybody, will be nice.
